# PERSONALITY AND ALLOSTATIC LOAD: TESTING HEALTHY NEUROTICISM IN HISPANIC AMERICANS OVER 50

**DOI:** 10.1093/geroni/igae098.2235

**Published:** 2024-12-31

**Authors:** Barış Sevi, Adil Supiyev, Angela Gutierrez, Graciela Muniz Terrera

**Affiliations:** MEF University, Istanbul, Istanbul, Turkey; Ohio University Heritage College of Osteopathic Medicine, Athens, Ohio, United States; Western University of Health Sciences, Pomona, California, United States; Ohio University, Athens, Ohio, United States

## Abstract

Allostatic load (AL) measures the cumulative wear and tear resulting from chronic stress and life events. Hispanic populations report a higher risk for elevated AL. To diminish health inequities in socially disadvantaged populations, understanding the antecedents of AL is essential. Using data from the Health and Retirement Study, this study examines the Big-5 personality traits as predictors of AL among middle-aged and older Hispanic adults (age ≥ 50; n: 319). First, a conditional growth model was tested where baseline Big Five personality traits were entered simultaneously to test the association with AL over three time points in eight years (Time 1 = 2006/2008; Time 2 = 2010/2012; Time 3 = 2014/2016). Our findings indicate that higher conscientiousness at baseline was associated with lower AL, but the Big-5 personality traits did not significantly predict the rate of change in AL over time. As the healthy neuroticism hypothesis suggests that higher neuroticism, which is by itself associated with worse health, when combined with higher conscientiousness, may positively influence health outcomes, a second conditional growth model was tested with the interaction of neuroticism and conscientiousness added to the first model. Contrary to expectations, higher levels of neuroticism and conscientiousness were associated with higher baseline AL, with no significant association with the rate of change in AL. These results underscore the importance of personality in predicting AL but provide evidence that challenges the assumptions of the healthy neuroticism hypothesis.

